# Supplementary material for: Effectiveness of digital interventions to improve household and community infection prevention and control behaviours and to reduce incidence of respiratory and/or gastro-intestinal infections: a rapid systematic review
Source: BMC Public Health. 2021 Jun 21;21:1180. doi: 10.1186/s12889-021-11150-8 (PMC8215628; doi:10.1186/s12889-021-11150-8)
Supplement: Supplementary file 1 — Additional file 1. Full list of search terms. Search strategy in digital databases. [file 12889_2021_11150_MOESM1_ESM.docx]

Database: Ovid MEDLINE(R) and Epub Ahead of Print, In-Process & Other Non-Indexed Citations, Daily and Versions(R) <1946 to May 19, 2020>

Search Strategy:

--------------------------------------------------------------------------------

***IPC terms***

1 Hand Disinfection/ (5555)

2 Hand Disinfection/ (5555)

3 Infection Control/ (23691)

4 (hand* adj3 wash*).ti,ab,kw. (4136)

5 "hand hygiene".ti,ab,kw. (4299)

6 (infect* adj3 prevent*).ti,ab,kw. (40189)

7 (infect* adj3 control*).ti,ab,kw. (52965)

8 (infect* adj3 risk*).ti,ab,kw. (57823)

9 (infect* adj3 transmiss*).ti,ab,kw. (14062)

10 (virus* adj3 prevent*).ti,ab,kw. (4070)

11 (hand* adj3 saniti?er).ti,ab,kw. (266)

12 (hand* adj3 saniti?ing).ti,ab,kw. (52)

13 (facemask* or "face mask*").ti,ab,kw. (4327)

14 Masks/ (4246)

15 (disinfect* adj3 surface*).ti,ab,kw. (1247)

16 Disinfection/ (14521)

17 (PPE or "personal protective equipment").ti,ab,kw. (5441)

18 Personal Protective Equipment/ (574)

19 or/2-18 (201481)

***Digital intervention terms***

20 Internet/ (72192)

21 Online Social Networking/ (136)

22 Telemedicine/ (22108)

23 Internet-Based Intervention/ (104)

24 Smartphone/ (4210)

25 Cell Phone/ (8419)

26 Social Media/ (7509)

27 Video Games/ (5326)

28 Mobile Applications/ (5670)

29 Text Messaging/ (2797)

30 Virtual Reality/ (1761)

31 Computer Simulation/ (186813)

32 Wearable Electronic Devices/ (2308)

33 Social Networking/ (3313)

34 Health Behavior/ (49433)

35 or/20-34 (352709)

36 intervention*.ti,ab,kw. (980781)

37 35 and 36 (32574)

38 (digital* adj3 intervention*).ti,ab,kw. (901)

39 (online adj3 intervention*).ti,ab,kw. (1630)

40 (mobile* adj3 intervention*).ti,ab,kw. (1520)

41 (smartphone* adj3 intervention*).ti,ab,kw. (328)

42 (cellphone* adj3 intervention*).ti,ab,kw. (4)

43 (mhealth adj3 intervention*).ti,ab,kw. (891)

44 (ehealth adj3 intervention*).ti,ab,kw. (521)

45 (app-based adj3 intervention*).ti,ab,kw. (126)

46 (website* adj3 intervention*).ti,ab,kw. (211)

47 (web-based adj3 intervention*).ti,ab,kw. (1753)

48 (internet adj3 intervention*).ti,ab,kw. (1706)

49 (computer-based adj3 intervention*).ti,ab,kw. (379)

50 (social media adj3 intervention*).ti,ab,kw. (269)

51 (video game* adj3 intervention*).ti,ab,kw. (77)

52 (text messag* adj3 intervention*).ti,ab,kw. (666)

53 (gaming adj3 intervention*).ti,ab,kw. (57)

54 (sms messag* adj3 intervention*).ti,ab,kw. (13)

55 (mobile messag* adj3 intervention*).ti,ab,kw. (6)

56 (electronic* adj3 intervention*).ti,ab,kw. (708)

57 (virtual reality adj3 intervention*).ti,ab,kw. (211)

58 (mobile app adj3 intervention*).ti,ab,kw. (77)

59 (phone app adj3 intervention*).ti,ab,kw. (15)

60 (smart electronic device* adj3 intervention*).ti,ab,kw. (0)

61 (wearable* adj3 intervention*).ti,ab,kw. (69)

62 (online coach* adj3 intervention*).ti,ab,kw. (2)

63 (online network* adj3 intervention*).ti,ab,kw. (0)

64 or/37-63 (37764)

65 19 and 64 (598)

66 limit 65 to yr="2000 -Current" (533)

Database: China National Knowledge Infrastructure <1^st^ Jan 2000 to 22^nd^ May 2020>

Search Strategy:

*--------------------------------------------------------------------------------*

***IPC terms***

1. SU='消毒'+'洗手'+'手卫生'+'手部卫生'+'控制感染'+'预防感染'+'预防病毒'+'预防细菌'+'预防病菌'+'感染风险'+'传染'+'口罩' (144,886)

***Digital intervention terms***

1. SU='远程医疗'+'远程健康'+'远程患者监测'+'互联网医疗'+'线上医疗'+'在线干预'+'线上干预'+'在线医疗'+'数字干预'+'数字疗法'+'数字治疗'+'数字医疗'+'行为干预'+'行为改变'+'行动干预'+'应用程序干预'+'app'+'Mhealth'+'ehealth'+'智能手机'+'社交媒体'+'电子游戏'+'移动应用程序'+'短信'+'虚拟现实'+'计算机模拟'+'可穿戴电子设备'+'社交网络'+'健康行为'+'微信'+'微博'+'QQ'+'论坛' (3,156,944 )
2. 1 AND 2 (1310)
