# Supplementary material for: Effectiveness of digital interventions to improve household and community infection prevention and control behaviours and to reduce incidence of respiratory and/or gastro-intestinal infections: a rapid systematic review
Source: BMC Public Health. 2021 Jun 21;21:1180. doi: 10.1186/s12889-021-11150-8 (PMC8215628; doi:10.1186/s12889-021-11150-8)
Supplement: Supplementary file 3 — Additional file 3. Table of included study characteristics. Summary of studies included in the systematic review sorted by population, sample size, intervention(s), control, outcome measures and results. [file 12889_2021_11150_MOESM3_ESM.docx]

| Study | Country | Design | Disease | Population | Population characteristics | Sample Size | Setting | Mode of Delivery | Intervention(s) | Control | Duration of intervention | Outcome Measures and when they were taken | Results |
| --- | --- | --- | --- | --- | --- | --- | --- | --- | --- | --- | --- | --- | --- |
| Bourgeois 2008 | US | RCT | Flu | Healthy adults | Intervention:  58% female  Mean age 46.4  Control:  37% female  Mean age 46.9 | 144 employees from across 8 sites  registered for the study and 125 completed the baseline survey. Of these, 119 (95%) completed between  one and seven biweekly surveys, and 99 (79%) completed the exit survey. | Workplace | Personally controlled health record program:  Enrolled subjects completed online health risk assessment surveys, the responses  to which drove the decision support system to generate and send tailored health messages for participants  in the intervention group. These messages were sent to participants’ PING record inbox, and participants  were simultaneously notified with a standard, plain-text email instructing them to visit and log on to their  PING record to review the message. | Messages on influenza illness and prevention, tailored based on information provided in baseline and seven bi-weekly surveys, and on postcode. There were five types of health message: vaccine reminders, respiratory illness advice, influenza alerts, weekly influenza risk maps, monthly educational bulletins. | Four bulletins, sent monthly, providing information on cardiovascular disease, stroke, skin  cancer and sun protection, and guidelines for a healthy diet. | 16 weeks | Measurements of behavior change included:  the rate of work attendance despite a respiratory illness, and responses to two questions in the baseline and exit surveys on hand hygiene and cough etiquette.  The exit survey was completed at the end of the intervention period. | Participants in the intervention group were more likely to stay home during an infectious respiratory illness compared with participants in the control group (39% [16/41] vs 14% [5/35], respectively; *P* = .02).  The two questions addressing hand hygiene and cough etiquette did  not show any changes in behavior among the intervention group. |
| Hu 2018 | China | Non-randomized but controlled trial | HFMD | Staff and parents in two kindergartens of the South Gate community health service centre | Intervention:  Children:  47% female  Mean age 4.9  Parents:  43% female  Mean age 32.9  Daycare staff:  100% female  Mean age 23.1  Control:  Children:  53% female  Mean age 5.2  Parents:  40% female  Mean age 29.5  Daycare staff:  100% female  Mean age 22.8 | 130 (10 daycare staff, 60 children and 60 parents) | Behaviour in kindergartens and home | WeChat | WeChat education (HFMD) + Usual care:  In addition to usual health education, GP, daycare staff and parents establish WeChat group to circulate health education knowledge on HFMD prevention. The main contents of health education included knowledge on HFMD, hand washing, health behaviour cultivation, supervising and urging the kindergartens and children's families to do a good job in environmental sanitation, morning inspection, etc. | Usual care (health education): Usual health education is provided by setting up bulletin boards, conducting regular health education lectures and distributing publicity materials. This is delivered face to face. | 3 months | Incidence of HFMD  Proportion of children who mastered the correct way of washing hands  Proportion of children who formed good habits of washing hands  The measurement time point was end of treatment. | Incidence of HFMD: Intervention= 0% [0/30]; Control=13.3% [4/30].  Proportion of children who mastered the correct way of washing hands: Intervention= 96.67% [29/30]; Control=76.67% [23/30]; p<0.05.  Proportion of children who formed good habits of washing hands: Intervention= 96.67% [29/30]; Control=66.67% [20/30]; p<0.05. |
| Hu 2019 | China | RCT | HFMD | Parents of children with HFMD | Intervention:  40% female  Mean age 4.9  Control:  43% female  Mean age 5.2 | Parents of 120 children | South Gate Community Health Service Center and home from March 2017 to October 2017 | WeChat | The GP team and the parents establish a WeChat group for health education, in which the GPs upload the professional knowledge and nursing methods of HFMD, and the recognition knowledge of severe cases. Push classic cases of HFMD, including children's psychological care, life care, symptomatic care and drug care. Accept the knowledge consultation of HFMD, answer the questions raised by the parents, so that the parents in the group can grasp the most basic medical information of HFMD. Provide timely treatment and nursing services when necessary. | Usual health education, including introduction of disease knowledge, treatment and nursing, diet and medication, disinfection and isolation, etc., and distribution of education materials. Provided by GP face to face. | 14 days | Duration of rash  Time to recovery  The measurement time point was end of treatment. | Duration of rash M (SD):  Intervention= 3.65 (0.8), control 7.43 (1.9)  Time to recovery M (SD):  Intervention = 6.66 (1.5), control 13.04 (2.6) |
| Judah 2009 | England | RCT | n/a (hand washing behaviour, did not target specific disease; motivating diseases were diarrheal diseases, prevent respiratory infections,  viral  infections, including norovirus, rotavirus, and  influenza, hospital acquired infections) | Highway service station bathroom users | Since it was the summer holidays, it would have been a wide spectrum of the travelling public, including families, young people, the elderly, as well as weekday business clientele | 32 days of data, measuring more than 108000 male restroom uses and more than 90000 female restroom uses | Service station bathrooms | Text-only messages (max 48 characters) displayed on an electronic dot matrix  screen over the entryway to the 2 restrooms; messages were in  capital letters, and they flashed for the duration  of their presentation to attract attention (except  the blank control condition, when no message  was displayed). | Intervention messages covered seven domains and all included the word ‘soap’. Two messages were tested for each domain. The domains were: Knowledge of risk, Inform people about a fact they may not know; Knowledge activation. Remind people of  what they know already or convince them of  the importance of what they know; Norms or affiliation. Raise concern for social  judgments on people’s hygiene behaviors  because of the knowledge that others might  be concerned with standards for acceptable  behavior; Status or identity, Help people to feel that  hand washing—or more broadly, cleanliness  and being hygienic—is an important aspect of their self-image; Comfort. Emphasize positive sensory qualities  of having clean hands; Disgust. Trigger the arousal of a ‘‘yuck’’  response; Cue. Provide people with a behavioral rule  triggered by an object in the environment or  an event. | Blank control: no message on the board  Positive control: ‘‘Wash your hands with  soap.’’ | July-September 2008 | Soap use ratio: Soap dispenser use divided by number of people in the bathroom during the intervention period (I-hour intervals) | Most domains showed a small but significant increase in soap-use ratio when compared with  the blank control; however, the pattern of results was very different for men and women.  For men, disgust and norms were the most effective (with disgust corresponding to a 9.8%  relative increase compared with the control; P=.001), followed by status, positive control, cue, and comfort; knowledge of risk and knowledge activation were the least effective.  For women, all domains showed an increase; however, some were not significantly better than the control condition. Knowledge activation, positive control, and knowledge of  risk were the most effective (with knowledge activation corresponding to a 9.4% increase  compared with the control condition; P=.001), followed by norms and status. Disgust  and cue were only slightly better than the control condition, and comfort only marginally so.  The only message that was effective for both genders was the norms message, ‘‘Is the person next to you washing with soap?’’, which resulted in a 12.1% relative increase in hand-washing ratio among men and a 10.9% increase among women compared with the control condition. |
| Little 2015 | UK | RCT | RTIs | Adult patients (aged 18 years or older) identified from computerised lists in general practitioner (GP) practices in England, for whom there was at least one other individual living in the household who was willing to report illness to the index person. | Intervention  56%  Age 56.7  Control  Women 56%  Age 56.5 | 20,066 in 344 practices | Recruitment from GP practice, trial online | Web-based and email | There were four weekly web-based sessions, each with new content to encourage participant interest and to maximise retention. The intervention provided  information about the importance of influenza and the role of handwashing, developed a plan to maximise intention formation for handwashing, reinforced helpful attitudes and norms, and addressed negative beliefs and used tailored feedback. Automated emails were used to prompt participants (to use sessions, to complete the monthly questionnaires, and in the intervention group questions on a monthly basis to maintain handwashing). | No treatment: The control group did not have access to the intervention webpages, but they had monthly emails to prompt them to complete the questionnaires. Similar to the intervention group, the control group had access to the GP practice in the normal way for respiratory illnesses. | 4 months | Number of index cases reporting RTI at 16 weeks  Number of respiratory infections in a household member in past 4 months  GI in the last 4 months  Number of respiratory infections in past 4 months  Number of respiratory infections in the household in the past 4 months  Number of days of moderate or bad symptoms in all those who had an infection  Number of index individuals who reported one or more RTIs at 16 weeks  Secondary outcomes:  Duration of symptoms  Transmission of respiratory infections  Gastrointestinal symptoms  Attendance at the practice  Use of health services | After 16 weeks, 4242 individuals (51%) in the intervention group reported one or more episodes of RTI compared  with 5135 (59%) in the control group (multivariate risk ratio 0·86, 95% CI 0·83–0·89; p<0·0001). The intervention  reduced transmission of RTIs (reported within 1 week of another household member) both to and from the index  person. We noted a slight increase in minor self-reported skin irritation (231 [4%] of 5429 in intervention group *vs*  79 [1%] of 6087 in control group) and no reported serious adverse events. |
| Tidwell 2019  Study 2 | India:  Varanasi, Allahabad, and Gorakhpur in the northern Indian state of Uttar Pradesh—less wealthy areas | RCT with prospective randomisation and retrospective matching | n/a (hand washing behaviour, did not target specific disease; motivating diseases were diarrheal disease,  rates of acute  respiratory infection, eye infections, and school absenteeism) | New mothers and mothers of young children (4-7 years old) | Modal age in both groups was 25-9 | New mothers: 419 enrolled in the intervention  arm (367, 87.6% retained overall), 198 in the control  General mothers, 371 enrolled in the intervention arm (303, 81.7% completed the study), 234 in the control | Home | Mobile phone audio messages for all mothers; reminder texts for new mothers | Mobile phone Messages: Mothers received audio phone messages twice  weekly for 8 weeks (new mothers) or 4 weeks (mothers of 4–7 year-olds).  Messages to general  mothers were about hygiene topics, while messages to new  mothers included more general maternal health messages as  well.  Messages were rendered  through stories involving a fictionalized local doctor who represented  an authority figure and was well versed in local health  beliefs. Messages were about 90 seconds long and included  a jingle to identify and brand the campaign, a dialogue between  the doctor and a mother, and then a summary and sign off  directed back to the caller.  New mothers also received reminder texts  to practice the target behaviors. | No intervention (though control sample also had to own a phone) | 8 weeks  New mothers for eight weeks and mothers of 4-7 year-olds for four weeks. | Handwashing with soap at key occasions, measured 21 days after the end of the intervention (using sticker diaries, which consisted of a blank page  divided into seven parts to represent times of day; participants put stickers in chronological order  within each section to correspond with recalled behaviors, they were also asked to recall distractor behaviours, to mask the purpose of the study). | New mothers were more likely to wash their hands than the control group (adj-RR: 1.04, p = .035), corresponding to 1.3 more occasions daily, and a 3.0 percentage point increase from  a baseline rate of 49.6%  General mothers were more likely to wash their hands than the control group (RR: 1.07, p = .007), corresponding to 1.0  more occasions daily, and an increase of 3.4 percentage  points over a baseline rate of 46.7%.  As the base rates were 8.8  handwashes with soap at key times in the new mothers’ intervention  and 6.8 for general mothers, this corresponds to an increase to  a mean of 10.1 handwashes per day for new mothers and 7.8 for  general mothers, a 15% relative increase in each group. |
| Wu 2020 | China | RCT | HFMD | Parents of healthy children aged 0-3  Vaccinated | Intervention:  46% female  Mean age 34.6  Control:  48% female  Mean age 34.2 | 3000 | Home | Text messages | text messages of < 50 words, about knowledge, prevention and treatment of HFMD, sent at least weekly starting one month before the peak time for HFMD (April-July, so first message in March), with a  total of 16-20 messages. | No intervention | 5 months | Proportion of children who wash hands before eating and after going to the toilet  Proportion of children who wash hands after going out  (Both measured at baseline and at one year) | Proportion of children who wash hands before eating and after going to the toilet: intervention= 93.6% [1300/1389]; Control=71.7% [987/1376]; p<0.01.  Proportion of children who wash hands after going out: intervention= 92.6% [1286/1389]; Control=69.1% [951/1376]; p<0.01. |
